# Genetic Variants of Angiotensin-Converting Enzyme Are Linked to Autism: A Case-Control Study

**DOI:** 10.1371/journal.pone.0153667

**Published:** 2016-04-15

**Authors:** Negar Firouzabadi, Nima Ghazanfari, Ali Alavi Shoushtari, Nasrallah Erfani, Farshid Fathi, Mozhdeh Bazrafkan, Ehsan Bahramali

**Affiliations:** 1 Department of Pharmacology & Toxicology, School of Pharmacy, Shiraz University of Medical Sciences, Shiraz, Iran; 2 Department of Pharmacology, School of Pharmacy, Shiraz University of Medical Sciences, International Branch, Shiraz, Iran; 3 Department of Psychiatry, School of Medicine, Hafez Hospital, Shiraz University of Medical Sciences, Shiraz, Iran; 4 Cancer Immunology Group, Shiraz Institute for Cancer Research, School of Medicine, Shiraz University of Medical Sciences, Shiraz, Iran; 5 Department of Speech Therapy, School of Rehabilitation, Shiraz University of Medical Sciences, Shiraz, Iran; 6 Noncommunicable Diseases Research Center, Fasa University of Medical Sciences, Fasa, Iran; Ehime University Graduate School of Medicine, JAPAN

## Abstract

**Background:**

Autism is a disease of complex nature with a significant genetic component. The importance of renin-angiotensin system (RAS) elements in cognition and behavior besides the interaction of angiotensin II (Ang II), the main product of angiotensin-converting enzyme (ACE), with neurotransmitters in CNS, especially dopamine, proposes the involvement of RAS in autism. Since the genetic architecture of autism has remained elusive, here we postulated that genetic variations in RAS are associated with autism.

**Methods:**

Considering the relation between the three polymorphisms of ACE (I/D, rs4343 and rs4291) with the level of ACE activity, we have investigated this association with autism, in a case-control study. Genotype and allele frequencies of polymorphisms were determined in DNAs extracted from venous blood of 120 autistic patients and their age and sex-matched healthy controls, using polymerase chain reaction (PCR) and PCR–restriction fragment length polymorphism (PCR–RFLP) methods.

**Results:**

There were strong associations between both DD genotype of ACE I/D and the D allele, with autism (P = 0.006, OR = 2.9, 95% CI = 1.64–5.13 and P = 0.006, OR = 2.18, 95% CI = 1.37–3.48 respectively). Furthermore, a significant association between the G allele of rs4343 and autism was observed (P = 0.006, OR = 1.84, 95%CI = 1.26–2.67). Moreover, haplotype analysis revealed an association between DTG haplotype and autism (P = 0.008).

**Conclusion:**

Our data suggests the involvement of RAS genetic diversity in increasing the risk of autism.

## Introduction

Autism is a neuropsychiatric disability, characterized by impairments in social interaction and communication and also by restricted repetitive and stereotyped patterns of behavior [1]. A survey in 2014 revealed the overall prevalence of autism to be 14.7 per 1,000 (one in 68) in children aged 8 years in the United States [2]. The prevalence of autism in Iran was estimated to be 6.2 per 1,000 in children aged 5 years [3]. Literature survey shows a marked male preponderance, with the male-to-female ratio of about 4:1 in autistic patients [4]. While the exact etiology of autism remains unknown, the significant role of genetics is not negligible.

Several lines of evidence suggest that autism is one of the most heritable neuropsychiatric disorders [5]. Family studies have shown a sibling prevalence risk of 2%-6% which is remarkably higher than that in general population [6]. A number of candidate genes assumed to be involved in the pathophysiology of autism, have been proposed in association studies in the last few years [7–12] among which the neurotransmitter system was of great attention. Probable dysfunction of the dopamine system in the pathogenesis of autism is frequently reported [9, 13]. Serum level of homovanillic acid, the main dopamine metabolite, is shown to be elevated in the cerebrospinal fluid of autistic patients [14]. Moreover, most autistic children are also diagnosed with attention-deficit hyperactivity disorder (ADHD) [15]. Dysfunction of the dopaminergic system contributes substantially to the etiology of ADHD [16, 17]. According to a recent report, 31% of autistic children take an antipsychotic medication [18] among which risperidone is vastly prescribed. Risperidone was shown to be well effective in treating aggressiveness, hyperactivity, irritability, self-injurious, stereotypic behavior, social withdrawal and lack of interest [19, 20]. Pharmacological efficacy of this drug is primarily initiated by dopamine receptor blockade [21] which further supports the role of dopamine in pathophysiology of autism.

Renin angiotensin system (RAS) has been hypothesized to have pivotal role in some psychiatric and neurological diseases [22–28]. Angiotensin-converting enzyme (ACE) is the essential enzyme in this system and catalyzes the conversion of angiotensin I (Ang I) to Ang II. ACE also plays a major role in the degeneration of neurokinins, a family of neurotransmitters in the central nervous system (CNS). The implication of neurokinins in psychiatric disorders is supported by their function in regulation of emotions, cognition, behavior and memory [29–32] which are disrupted in autism. [33, 34]. Ang II, the ultimate product of RAS, is also assumed to interact with neurotransmitters such as dopamine and serotonin in CNS which proposes a possible mechanism of action for Ang II in behavioral and cognitive processes [35, 36]. Brain Ang II has been proposed to induce dopaminergic cell death via production of reactive oxygen species (ROS) [37]. Besides a loss of dopamine secreting capacity, the resultant neuro-inflammatory ramifications are thought to be involved in autism as well as other neurodevelopmental conditions [38, 39]. Ang II, with pro inflammatory characteristics, exerts most of it physiological action via two main receptors of angiotensin II type 1 and type 2 receptor which have been found to be widely distributed in different areas of the brain associated with cognitive functions [40] including areas affected in autism.

Activity of RAS is governed by genetic determinants in a variety of ways. It has been suggested that several single nucleotide polymorphisms (SNPs) on the ACE gene such as rs4343, rs4291 and also ACE I/D, determine the activity of this enzyme and the level of Ang II [27, 41].

Considering the complexity of autism’s etiology and multiple parameters being involved in this disease, of which genetic factors are more significantly involved, in this case control study, for the first time, we have evaluated the association of three genetic variants of the ACE gene (I/D, rs4343, rs4291) with autism.

### Methods

One hundred and twenty outpatient autistic children (86 males, 34 females) age of 3 to 12 years old (mean ± S.D: 7.5±2.8) with a clinical diagnosis of autism (DSM-IV-TR) and 120 age and sex matched healthy control cases (86 males, 34 females) from Imam Reza Hospital, Shiraz, Iran were included in the study between the years 2012–2014. Healthy control subjects were from the same geographical area as our patient groups. All individuals in control group were behaviorally and somatically healthy. They had no history of psychiatric disorders and never took medications for psychiatric conditions. This work was carried out in accordance with The Code of Ethics of the World Medical Association (Declaration of Helsinki) and Uniform Requirements for manuscripts submitted to biomedical journals. The study was approved by the local committee of ethics of medical experiments on human subjects of Shiraz University of Medical Sciences. Informed written consent (approved by The Institutional Ethical Committee) was obtained from parents/legal representatives of all individual participants included in the study. Diagnosis of autism was based on DSM-IV-TR criteria, ASD Diagnostic Interview-Revised [42], expert clinical evaluation; Clinical Global Impressions-Severity (CGI-S) rating of 4 or greater [43] and Autism Behavior Checklist (ABC) [44].

### Genotyping

Genomic DNAs were extracted from whole blood leukocytes, using the salting out method [45]. The extracted DNAs were dissolved in sterile distilled water and stored at 4°C for further PCR analysis. PCR amplification/detection of ACE I/D was carried out using a standard protocol [46]. In order to avoid mistyping of ID as DD genotype, all DD genotypes were reconfirmed by another typing system [47]. PCR amplification of rs4291 and rs4343 was performed as described in detail previously by Firouzabadi et al. [48]. It is to mention that all of the samples were genotyped at least twice and reconfirmed.

### Statistics

The data were analyzed using SPSS® 22.0 for windows® (SPSS Inc.,Chicago, Illinois). All continuous variables are presented as mean ± S.D. Genotype frequencies are expressed in percentage (%). Hardy–Weinberg equilibrium (HWE) for the distributions of genotypes was estimated by chi-square (χ2) test. Distribution of all continuous variables was tested for normal distribution with the Kolmogorov–Smirnov test.

Pearson's chi-square or Fisher's exact test were applied to assess differences in the genotype and allelic (respectively) distribution between groups of patients and controls. Pair-wise comparisons were performed by Bonferroni’s post-hoc test. Odds ratio (OR) and 95% confidence intervals (CI) were obtained. P value <0.05 was considered as statistically significant. Relationship between different haplotypes and autism was calculated using Arlequin 3.1 software package [49].

## Results

One hundred and twenty children with a diagnosis of autism were molecularly genotyped for ACE I/D and two SNPs within the ACE gene (rs4291 and rs4343). Genotypic and allelic distributions were compared to those obtained from 120 age and sex-matched healthy subjects of Caucasian origin (86 males, 34 females). Genotypes were in Hardy-Weinberg Equilibrium in control individuals (P-value for rs4291, rs4343, ACE I/D = 0.391, 0.965, 0.343 respectively). Table 1 shows genotype distribution, allele frequencies and associations between study variants and autism. GG genotype of rs4343 was more frequent in autistic patients (P = 0.012, OR = 1.97, 95%CI (1.16–3.32); however after applying Bonferroni's correction this association was not significant (adjusted P = 0.069). In addition, G allele of rs4343 was significantly associated with autism after using Bonferroni's correction [adjusted P = 0.006, OR = 1.84, 95%CI (1.26–2.67) respectively]. On the other hand, DD genotype of ACE I/D as well as D allele of this variant revealed a significant association with autism [adjusted P = 0.006, OR = 2.9, 95% CI (1.64–5.13) and adjusted P = 0.006, OR = 2.18, 95% CI (1.37–3.48) respectively]. Regarding haplotype analysis (Table 2), DTG haplotype was significantly higher in autistic patients comparing with healthy controls (adjusted P = 0.008). Moreover, IAA haplotype was significantly higher in healthy subjects vs. patients group (adjusted P = 0.047).

**Table 1:** Genotype distribution and allele frequencies in depressed patients and healthy controls.

Variables	Autistic patients N (%)	Healthy controls N (%)	P-value	Adjusted P-value*	OR (95%CI)
rs4291			0.033	0.182	0.56 (0.33–0.96)
AA	38 (31.6%)	54 (45%)			
AT	77 (64.2%)	56 (46.7%)			
TT	5 (4.2%)	10 (8.3%)			
Alleles			0.335	0.913	1.23 (0.84–1.79)
A	153(63.75%)	164 (68.3%)			
T	87 (36.25%)	76 (31.7%)			
rs4343			0.012	0.069	1.97 (1.16–3.32)
AA	37 (30.8%)	58 (48.3%)			
AG	59 (49.2%)	51 (42.5%)			
GG	24 (20%)	11 (9.2%)			
Alleles			0.001	0.006	1.84 (1.26–2.67)
A	133 (55.4%)	167(69.6%)			
G	107(44.6%)	73 (30.4%)			
ACE I/D			0.001	0.006	2.9 (1.64–5.13)
II	8 (6.7%)	10 (8.3%)			
ID	17 (14.2%)	42 (35%)			
DD	95 (79.2%)	68 (56.7%)			
Alleles			0.001	0.006	2.18 (1.37–3.48)
I	33 (13.75%)	62 (25.8%)			
D	207 (86.25%)	178(74.2%)			

*: P-value after applying Bonferroni's post-hoc test.

**Table 2:** Comparison of haplotype frequencies in autistic patients and healthy individuals.

ACE I/D	rs4291	rs4343		Case group			Control group		P-value	Adjusted P-value*
D	T	G	+	57	23.7%	+	33	13.7%	0.001	0.008
			-	183	76.3%	-	207	86.3%		
D	A	A	+	83	34.6%	+	86	35.8%	0.670	0.426
			-	157	65.4%	-	154	64.2%		
I	A	A	+	24	10.0%	+	43	17.9%	0.006	0.047
			-	216	90.0%	-	197	82.1%		
D	A	G	+	41	17.1%	+	26	10.8%	0.031	0.222
			-	199	82.9%	-	214	89.2%		
I	T	G	+	4	1.7%	+	5	2.1%	1.000	1.000
			-	236	98.3%	-	235	97.9%		
D	T	A	+	26	10.8%	+	33	13.7%	0.294	0.938
			-	214	89.2%	-	207	86.3%		
I	A	G	+	5	2.1%	+	9	3.7%	0.271	0.920
			-	235	97.9%	-	231	96.3%		
I	T	A	+	0	0%	+	5	2.1%	0.061	0.396
			-	240	100%	-	235	97.9%		

*: P-value after applying Bonferroni's post-hoc test.

## Discussion

This is the first study examining the association of selected polymorphisms on ACE gene in a population of autistic children. The inspiration of the present study was the significant role of RAS in several neurological and psychiatric diseases [23, 24, 26, 27, 50] as well as in the ability of learning and memory [51, 52].

The novel findings of our study are the significant association of two polymorphisms, [ACE (I/D and rs4343)] located on ACE gene, with autism. The G allele of rs4343 increased the risk of autism by 1.84 fold versus carriers of the A allele (adjusted P = 0.006; OR = 1.84; 95%CI = 1.26–2.67). Another finding was the significant associations of DD genotype of ACE with autism. Inheritance of two D alleles increased the risk by 2.9 fold (adjusted P = 0.006; OR = 2.9; 95% CI = 1.64–5.13). Likewise, the D allele increased the risk by 2.18 fold (adjusted P = 0.006; OR = 2.18; 95% CI = 1.37–3.48).

As reported previously both alleles of D (ACE I/D) and G (rs4343) are assumed to be functional and increase serum ACE activity and as a result, produce higher levels of Ang II [41]. Furthermore, a recent report has shown a strong association of G allele of rs4343 with higher serum ACE concentration in a population of depressed patients. In the same study no association was observed between rs4291 and serum ACE activity [27]. The increased ACE activity may then explain the observed association of the mentioned polymorphisms with autism in our population. In addition, in our study population, DTG haplotype of study variants, was significantly higher in the patients group (adjusted P = 0.008) and IAA haplotype was significantly higher in the control group (adjusted P = 0.047) which suggests that the two variants of G (rs4343) and D (ACE I/D) may be linked to predisposition to autism, confirming the results obtained from genotype analysis.

There are several explanations for the involvement of RAS in autism. Among the proposed pathophysiological underpinning of autism, dysregulation of neurotransmitters function is consistently reported [9–11]. RAS is thought to interact with dopamine and serotonin in the brain and also alters neurokinins activity through ACE function [31, 32]. Neuro-inflammatory properties of Ang II may also describe the pathophysiological implications of RAS in autism [38, 39]. The pro inflammatory characteristics of circulating and tissue Ang II, is consistently reported to be involved in end organ damages after acute injuries like ischemia [53]. It is emphasized that blunting of RAS reduces the negative remodeling after myocardial infarction for example, and improves outcomes [54]. Similar consequences of local RAS activation in the brain may also explain the proposed role for RAS in cognitive and behavioral disorders. Brain is affected by systemic inflammation as well, like peripheral organs [55]. Patients with autism have been found to exhibit higher peripheral blood inflammatory biomarkers [56] which alter the permeability of the blood–brain barrier and may result in poor maintenance of the brain’s stability. Brain inflammation and the resultant damage [55], has major roles in the pathophysiology of various psychiatric and neurological disorders [57, 58].

Immune mediators such as interleukin-6 (IL-6) exert a regulatory function in all phases of central nervous system (CNS) development [59, 60]. Neurotropic properties of IL-6 are important in cell differentiation and axonal guidance in CNS [59, 61]. Alongside Ang II is considered a pleiotropic factor as well, secreted from most tissues including the brain [62]. Documents suggest that Ang II stimulates IL-6 synthesis and release. It is also demonstrated that Ang II and IL-6 are co-localized in atherosclerotic plaques, suggesting the potential immune modulatory role of Ang II in tissues [63]. Moreover dopamine synthesis is further induced in inflammatory conditions of the brain. IL-6 stimulates hypothalamus-pituitary-adrenal (HPA) axis and the release of catecholamines such as dopamine [64]. The over production of Ang II is also one of the major causes of HPA axis dysregulation which has been observed in autism [65].

Ang II mediates most of its physiological action via two main receptors: angiotensin II type 1 and type 2 receptors, vastly distributed in the areas of the brain associated with cognitive function [40] including areas affected in autism. Implication of angiotensin receptor inhibitors (ARBs) has shown potent central anti-inflammatory properties of these agents [58]. The suggested role of inflammation in autism alongside the neuro inflammatory and oxidative actions of Ang II on one hand, and the role of neurotransmitters in autism [9, 66] and their interactions with RAS on the other hand [37, 67], constitute the justifications of our hypothesis of the contribution of RAS in the development of autism. However, this is the first report of such associations and needs to be replicated in other populations. Moreover to the best of our knowledge, the suggested proposal regarding the role of RAS in pathophysiology of autism is first described here and further evidence would saturate the idea. Inspired by the overwhelming evidence on the serious involvement of RAS after tissue injuries, regulating the local immune response and the overall tissue homeostasis, we believe further research is guaranteed regarding the role of RAS in autism.

Heterogeneity in expression of phenotypes in autism and its etiology has hindered the search for specific genes being involved in biological mechanisms that underlie its behavioral symptoms. Genome wide association studies have not yet identified the polymorphisms in RAS to be involved in autism. However, etiology of autism as a multifactorial disorder with the complex interplay of genetic and enviromental effectors, needs to be viewed in a broader context. Activity of the major neurohormonal systems of human body like RAS is believed to be echoed in response to extrinsic and intrinsic insults. Genetic determinants of RAS activity then would be prominent after the unfavorable outcome, here autism, has been occurred and may modulate the disease course, complications and response to therapy. It is of great interest to find out the exact pathophysiological mechanisms underlying the involvement of RAS in autism, as a widely distributed system involved in the homeostasis of virtually all tissues. The present study may provide some supporting evidence then.

## Conclusions

To conclude, the present study indicates that ACE I/D and rs4343 polymorphisms can be a risk factor associated with autism and this highlights the role of RAS in this illness. However, further genetic studies in various ethnicities and populations are warranted.

Ethical approval: “All procedures performed in studies involving human participants were in accordance with the ethical standards of the institutional and/or national research committee and with the 1964 Helsinki declaration and its later amendments or comparable ethical standards.”
